# Role of diacylglycerol kinase in autophagy, ER biogenesis, and triterpene metabolism

**DOI:** 10.1080/27694127.2022.2105455

**Published:** 2022-07-26

**Authors:** Kalyani Sai Reju, Chaithra Priya S, Ravi Manjithaya, Venkata Rao Dk

**Affiliations:** a Biochemistry laboratory, CSIR-Central Institute of Medicinal & Aromatic Plants, Research Center, Allalasandra, GKVK (post), Bangalore – 560065, INDIA; b Autophagy lab, Molecular Biology and Genetics Unit, Jawaharlal Nehru Centre for Advanced Scientific Research (JNCASR), Jakkur, Bangalore-560064; c Academy of Scientific and Innovative Research (AcSIR), Sector 19, Kamla Nehru Nagar, Ghaziabad, Uttar Pradesh-201 002, INDIA

**Keywords:** Autophagy, diacylglycerol kinase, endoplasmic reticulum, ER stress, synthetic biology, target of rapamycin (TOR) complex, triterpene

## Abstract

*Saccharomyces cerevisiae* is widely used for producing various triterpenes by exploiting its native mevalonate/ergosterol pathway. Yeasts that accumulate phospholipids can produce more triterpenes. Our recent study demonstrated that a high phospholipid-accumulating yeast phenotype, as in *spt10Δ* yeast, results in increased endoplasmic reticulum (ER) biogenesis, resulting in ER expansion. However, the *spt10Δ* strain also exhibits high reticulophagy. Dgk1 (diacylglycerol kinase) is an important enzyme in lipid metabolism, which synthesizes phosphatidic acid (PA) by phosphorylating diacylglycerol (DG). We demonstrate that *spt10Δ* yeast with increased Dgk1 activity offer two desired results, (i) a highly expanded ER, due to redirection of the lipid pathway away from triglycerides towards phospholipid synthesis, increasing ER biogenesis; and (ii) decreased reticulophagy, by increasing the PA pool that activates TOR complex-mediated autophagy suppression. It was speculated that more ER-bound pathway enzymes can fit in the expanded ER, and the mevalonate-ergosterol pathway, being ER bound, might have higher activity. This was demonstrated by the co-expression of Dgk1 and plant triterpene synthase in *spt10Δ* yeast, which shows a high accumulation of plant triterpene.

## Phospholipids and ER biogenesis

Phospholipids (PLs) are synthesized by sequential acylation of glycerol-3-phosphate. The PLs play an important role in membrane biogenesis, cell signaling, and the development of subcellular organelles. PA is an anionic phospholipid that acts as a central molecule in phospholipid and neutral lipid biosynthesis. In *spt10* (a global transcription regulator) deletion yeast, *spt10Δ*, there is an increase in total lipids, resulting in the accumulation of both phospholipids and neutral lipids. The increased total phospholipids are directed to endoplasmic reticulum (ER) membrane growth. With fluorescence microscopy, we observe an increased concentration of ER, indicating a boost in ER biogenesis [1]. We also see proof of neutral lipid accumulation by the significant increase in lipid droplets (LDs). However, excess ER biogenesis and high LDs induce autophagy, leading to unstable ER structure and high ER stress.

## Elevated Dgk1 activity suppresses autophagy

**Figure 1.**
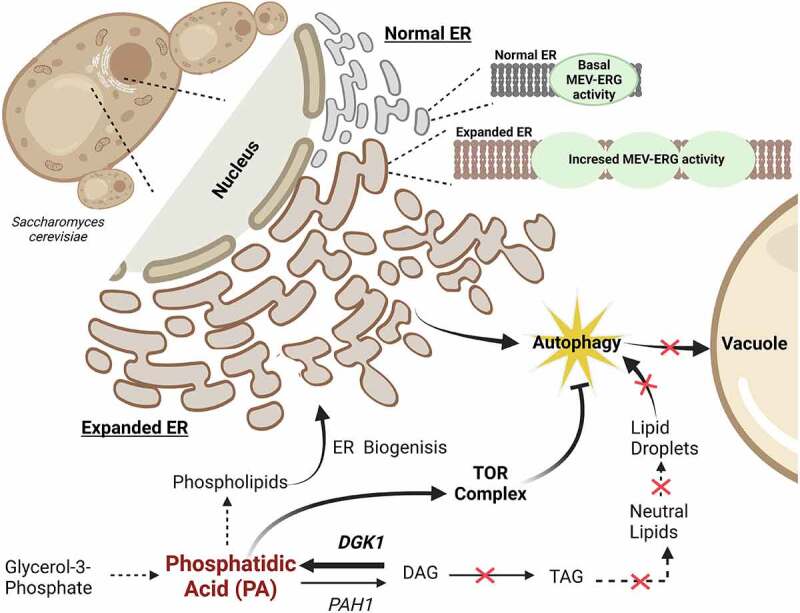
The schematic diagram demonstrates the link between the lipid pathway, endoplasmic reticulum (ER), and mevalonate-ergosterol (MEV-ERG) pathway. *DGK1* overexpression increases PA and decreases flow towards neutral lipid (i.e., TAG) synthesis, reducing lipid droplet accumulation, which is known to activate autophagy. The enhanced PA pool increases ER membrane biogenesis leading to enlarged ER. Additionally, *DGK1* overexpression activates the TOR complex, which prevents autophagy and stabilizes the ER. It is speculated that the enlarged ER contains more surface area that accommodates significant amounts of ER-embedded proteins, thus increasing the MEV-ERG pathway activity in *spt10Δ* Dgk1 OE yeast. The figure was created using BioRender software.

Dgk1 is a cellular enzyme that primarily catalyzes the phosphorylation of DG to PA. Pah1/PA phosphatase and Dgk1 together work to maintain lipid homeostasis between PA and DG. Dgk1 overexpression (OE) increases the PA pool, which increases the availability of PA for phospholipid synthesis. It was found that autophagic activity in the *spt10Δ* strain is reduced with Dgk1 OE. Here, two effects can be considered; (i) Dgk1 by itself has been reported to be a possible autophagy suppressor; (ii) Dgk1 overexpression redirects the DG away from neutral lipid synthesis towards PA accumulation; this reduces lipid droplet formation, which is another factor that is known to control the autophagic process (Figure 1). Our fluorescence studies revealed that Dgk1 OE results in a stable expanded endoplasmic reticulum with minimal ER stress. It was observed that increasing PA levels lower ER stress. Decreased mRNA levels of unfolded protein response (UPR) markers (*IRE1*, spliced *HAC1*) and reduced vacuole fission indicate the decreased susceptibility to ER stress in the Dgk1 OE *spt10Δ* strain. Additionally, treatment of Dgk1 OE yeast with rapamycin (a TOR complex inhibitor) restores suppressed autophagy, suggesting that high Dgk1 activity may increase PA that activates the TOR complex and causes the suppression of the autophagic process in the Dgk1 OE yeast strain (Figure 1) [1].

## Triterpene production and expanded endoplasmic reticulum

In yeast, plant triterpenes can be synthesized by redirecting the intermediate metabolites of the mevalonate-ergosterol (MEV-ERG) pathway. Triterpenes are pharmacologically active compounds widely used in therapeutics, food, and personal care. Our previous study reported a novel connection between PA levels and MEV-ERG pathway activity, and it was found that increased PA levels result in improved MEV-ERG pathway activity. Our study was designed to exploit PL-accumulating yeast for improved plant triterpene production. For this, we used the PL-accumulating yeast knockout *spt10Δ*. We speculated that the *spt10Δ* strain could be utilized to develop a yeast expression system optimized for improved triterpene production. Initial analysis into squalene and ergosterol (MEV-ERG pathway metabolites) levels of *spt10Δ* yeast shows an insignificant difference in the accumulation compared to wild type (WT). Interestingly, the mRNA levels of MEV-ERG pathway genes shows significantly higher expression in the *spt10Δ* strain. The results indicated possible interruption in MEV-ERG pathway activity. As the MEV-ERG pathway is ER embedded, we focused on monitoring ER morphology, and this allowed us to identify unstable ER in *spt10Δ* yeast, which have high levels of autophagy and ER stress. Remarkably, we observe a significant increase in squalene and ergosterol levels with Dgk1 OE in *spt10Δ* yeast that is relative to the high mRNA levels of MEV-ERG pathway genes. With these observations, we theorize that the stable and expanded ER associated with the *spt10Δ* Dgk1 OE yeast phenotype possesses a large ER surface area, thus improving its protein storage capacity. As the MEV-ERG pathway is ER bound, the increased surface area likely allows for more embedded enzymes, thus increasing the MEV-ERG pathway activity (Figure 1). To demonstrate the increased MEV-ERG pathway activity in *spt10Δ* Dgk1 OE yeast, we introduced the plant triterpene synthase gene, β*-amyrin synthase*, from *Artemisia annua*. The metabolite quantification analyses of *spt10Δ* Dgk1 OE yeast shows a significant accumulation of β-amyrin, a natural plant triterpene.

## Conclusion

Increased PLs might be involved in different cell signaling processes, causing global regulation of various biochemical pathways, including MEV-ERG pathway regulation. However, how exactly PLs regulate ER biogenesis and expansion has to be investigated further. Cell signaling and other factors involved in recruiting and synthesizing MEV-ERG pathway-specific enzymes on the ER membrane must also be investigated to improve the efficiency of triterpene synthesis. The autophagy restriction by Dgk1 OE opens a new avenue to retain an expanded and stable ER. The development of yeast phenotypes with stable expanded ER structure for target molecule production can be streamlined by further studies on how reticulophagy signals can be altered to recruit autophagy machinery. In our study, with a basic understanding of ER biogenesis through the regulation of phospholipid biosynthesis, a novel strategy is proposed to improve the commercial production of a triterpene, β-amyrin, in yeast. This increase in β-amyrin is caused solely by manipulating the phospholipid pathway without applying any traditional strategies used to increase triterpene production in yeast. The strategy needs to be optimized to work in tandem with existing MEV-ERG pathway manipulation strategies, which will undoubtedly give more promising results in the field of synthetic biology.
